# Management of topical steroid therapy in male patients with lichen sclerosus after circumcision

**DOI:** 10.3389/fmed.2026.1799218

**Published:** 2026-03-17

**Authors:** Lorenzo Pessina, Simone Ribero, Anna Verrone, Elena Stroppiana, Valentina Celoria, Pietro Quaglino, Luca Mastorino

**Affiliations:** Department of Medical Sciences, Dermatologic Clinic, University of Turin, Turin, Italy

**Keywords:** circumcision, DLQI, lichen sclerosus, phimosis, topical steroids

## Abstract

**Background:**

Lichen Sclerosus et Atrophicus (LSA) is a chronic inflammatory dermatosis of multifactorial aetiology, mainly affecting the genital area in both sexes and at any age. First-line therapy involves topical corticosteroids, whilst surgery, particularly circumcision in males, is reserved for non-responders or phimosis cases. Some patients show persistent disease post-surgery. The study aims to compare the effectiveness of circumcision versus topical corticosteroids in improving QoL in men with LSA, assess postoperative recurrence risk.

**Methods:**

A retrospective study of consecutive male patients with histological or clinical genital LSA were undergone. We collected clinical, anamnestic, and therapeutic data, including pre- and post-circumcision topical treatments. The DLQI questionnaire assessed quality of life according to treatment type.

**Results:**

Fifty-five males were analysed; 40% underwent circumcision. Of these, 83% used topical steroids before surgery and 68% resumed afterward (Steroid-Free Survival: 19 months). Resumption correlated with prior treatment (*p* = 0.043). QoL improved after circumcision and worsened with active therapy or phimosis (*p* = 0.002; *p* = 0.006).

**Conclusion:**

Circumcision improves QoL, especially in phimosis, though relapses are frequent. Topical therapies are commonly employed but do not appear to significantly impact QoL. The results underline that LSA management should be personalised, combining medical and surgical approaches based on severity and patient response.

## Introduction

Genital lichen sclerosus (LS), or lichen sclerosus et atrophicus (LSA), is a chronic inflammatory dermatosis of multifactorial origin, typically affecting the anogenital region (1).

LS arising from genetic predisposition, environmental factors, infections, hormonal changes, and immune system irregularities (2). Differently from women, in men, no clear genetic correlation has been observed, that suggests that LS’s inheritance patterns may differ by gender (2). Three primary mechanisms can be highlighted in LS pathogenesis: inflammation and activation of signalling pathways that lead to autoimmunity; formation of sclerotic tissue; and oxidative stress (2). Trauma and chronic irritation, such as occlusion, scratching, friction, or surgical procedures, can induce the Koebner phenomenon, where new LS lesions form at sites of skin injury (2). Some authors address a negative role of urine contact as irritating factor, especially in male patients with phimosis (3).

It occurs in both sexes and across a wide age range. In males, the disease often presents with phimosis, white atrophic plaques, erosions, and associated symptoms such as pruritus, dysuria, and sexual discomfort (1, 4). The disease can significantly impair quality of life (QoL) (5) and in some cases, may progress to squamous cell carcinoma (SCC) (6). The progressive nature of the pathogenesis accounts for the evolution of the lesions, which gradually lead to non-reducible phimosis with difficulty urinating during sexual intercourse in men, and progressive absorption of the external genital structures in women, with difficulty or even impossibility of sexual intercourse (2). First-line therapy includes potent topical corticosteroids (7, 8), sometimes combined with emollients or antioxidants like vitamin E (7). In refractory cases or when phimosis is present, circumcision is considered a definitive surgical intervention (1, 9). However, the persistence or recurrence of symptoms after circumcision has been observed in a subset of patients (10, 11), raising questions about long-term disease control and optimal treatment sequencing.

## Methods

This study aimed to assess the impact of different therapeutic approaches—namely circumcision and topical therapy—on the quality of life in male patients with genital LS. Additionally, the study explored recurrence patterns post-surgery, particularly the need to resume steroid therapy, and examined whether circumcision might confer a protective role against malignant progression to penile carcinoma.

This was a retrospective cohort study conducted on male patients with a clinical or histological diagnosis of genital LS followed at Genital Dermatology Clinic of AOU Città della Salute e delle Scienze di Torino. Patients were recruited based on clinical records, and relevant data were extracted regarding age, presenting symptoms, comorbidities, previous and ongoing treatments, and surgical history. Special attention was given to the use of topical corticosteroids and vitamin E prior to and after circumcision.

To assess quality of life, a cross sectional analysis was undergone, all consecutive patients completed the Dermatology Life Quality Index (DLQI), a validated, dermatology-specific instrument comprising ten items addressing symptoms, emotional well-being, daily activities, social interactions, and treatment burden (12). Patients reflected on their quality of life over the preceding 7 days and selected the response they considered most appropriate. Each response was scored as follows: “Not at all,” “Not relevant,” or “No” = 0; “A little” = 1; “A lot” = 2; and “Very much” or “Yes” = 3. The total DLQI score, obtained by summing individual item scores, ranges from 0 to 30, with higher scores indicating greater impairment in quality of life.

Statistical analyses included group comparisons using appropriate tests, such as the Chi-square test for categorical variables and the *t*-test for continuous variables. Effect size was assessed using Cohen’s *d*. Steroid-free survival after surgery was evaluated using the Cox proportional hazards regression model. A *p*-value <0.05 was considered statistically significant.

## Results

The study included 55 male patients, with a mean age of 66.07 years diagnosed with genital LS. Amongst these, 40% (22 pts) had undergone circumcision.

Preoperatively, 83% (18 pts) of these circumcised patients were already using topical corticosteroids, suggesting more severe or persistent disease. Postoperatively, 61% (13 pts) resumed corticosteroid therapy. The average Steroid-Free Survival post-circumcision was 19 months.

Importantly, the likelihood of restarting steroid treatment after surgery was significantly associated with prior corticosteroid use (*p* = 0.043). However, a Cox regression analysis excluded the presence of predictive factors (age, age at diagnosis, DLQI, pre-operatory application of vitamin E, pre-operatory application of steroids) for the resumption of topical steroid application in the postoperative period (Table 1).

**Table 1:** Demographic characteristics of the study population and steroid use in circumcised patients.

Variable	Value
General population (overall)	
Mean age, years	66.07
Mean age at diagnosis, years	57.8
Circumcised patients, %	40
Non-circumcised patients, %	60
Non-circumcised patients	
Indication for circumcision, %	24.24
No indication for circumcision, %	75.76
Circumcised patients (*n* = 100%)	
Application of topical steroids before circumcision, %	83.3
No application of topical steroids before circumcision, %	16.7
Post-operative topical steroid use (circumcised patients)	
Re-application of steroids post-operatively, %	61.1
No re-application of steroids post-operatively, %	38.9
Detailed distribution of circumcised patients	
Steroids before circumcision and re-application post-operatively, %	61.1
Steroids before circumcision without post-operative re-application, %	22.2
No steroids before circumcision with post-operative re-application, %	0
No steroids before circumcision and no post-operative re-application, %	16.7

Quality of life, as assessed by DLQI, showed significant improvement in circumcised patients compared to those under active topical therapy. Specifically, patients undergoing circumcision reported better overall QoL scores (*p* = 0,044), whilst those relying solely on medical management -particularly in the presence of phimosis- reported worse QoL (Table 2).

**Table 2:** DLQI in different groups of patients.

Group of patients	Number of patients	Mean score of DLQI	Standard deviation (DS)	*p*-value (undergoing circumcision)	*p*-value (indication for circumcision)
Undergoing circ.	22	2.41	4.49	0.044	–
No undergoing circ.	33	3.82	4.06	0.044	–
Planning circ.	8	6.00	6.35	–	0.234
No indication for circ.	25	3.12	2.85	–	0.234

Phimosis itself was significantly associated with both the indication for (*p* = 0.006) and the actual execution of circumcision (*p* = 0.002). As shown in Table 3, patients with phimosis were more likely to have undergone or be planned for circumcision compared to those without phimosis. Conversely, no statistically significant associations were found between any clinical or treatment-related variable and the incidence of penile carcinoma, suggesting that neither phimosis nor circumcision status was directly linked to the development of penile malignancy in this cohort.

**Table 3:** Associations of phimosis and circumcision with circumcision status and onset of penile carcinoma.

Variable	Group	*N* patients	Cases of carcinoma	%	*p*-value	Circumcision executed	*p*-value	Circumcision planned	*p*-value
Phimosis	Yes	34	2	5.9	0.613	55.9%	0.002	46.7%	0.006
	No	21	2	9.5		14.3%		6%	
Circumcision	Circumcised	22	2	9	1	–	–	–	–
	Not circumcised	33	2	6		–	–	–	–

## Discussion

This study contributes valuable insights into the clinical management of male genital LS. Circumcision was found to be beneficial in improving quality of life (13), particularly amongst those suffering from phimosis, a common and distressing manifestation of the disease. Nonetheless, the high rate of postoperative steroid resumption suggests that circumcision, whilst effective in relieving mechanical and anatomical symptoms, may not fully address the underlying inflammatory process in all patients.

These findings align with emerging literature that highlights the chronic-relapsing nature of LS and the need for long-term follow-up and treatment modulation (10).

The observed Steroid-Free Survival of 19 months represents a clinically relevant benchmark, providing objective guidance for clinicians in counselling patients on postoperative disease course and the expected duration of remission following circumcision.

The authors’ opinion is that, given the chronic-relapsing nature of the disease, completely discontinuing topical steroid therapy is not advisable, either pre- or post-operatively, for the purpose of ensuring the long-term success of the surgical procedure. The minimum dose of topical steroid necessary to prevent relapses should be maintained during. Early diagnosis is essential to prevent disease progression. Topical steroid treatments have been shown to be effective in limiting the progression of LS in both paediatric and adult-onset cases (14).

The lack of significant impact of topical therapy alone on QoL could be due to multiple factors: patients receiving conservative treatment may represent more severe or extensive disease (15), or they may have experienced suboptimal therapeutic response (8, 16). Additionally, the psychological and functional burden of untreated or partially treated phimosis likely contributes to lower QoL scores in this group (15).

Although this study did not reveal a significant association with penile carcinoma, these results should be interpreted with caution. The absence of neoplastic progression in the cohort does not rule out its potential occurrence, particularly considering the limited sample size and relatively short follow-up period. Furthermore, several studies have consistently shown that absence of circumcision represents a significant risk factor for the development of penile carcinoma (17). Vigilant surveillance remains essential, particularly in older patients or those with long-standing, refractory disease (18).

This study has several limitations. First, its retrospective design may introduce selection and reporting biases. The small sample size (*n* = 55) limits generalizability and reduces statistical power, especially for rare outcomes such as carcinoma. The use of self-reported DLQI questionnaires may also introduce recall bias, especially considering the cross-sectional approach, which is static and not dynamic. Moreover, the lack of a control group and standardised treatment protocols limits causal interpretation of treatment effects. Finally, the short median follow-up and heterogeneity in clinical presentations and therapeutic combinations complicate assessment of long-term outcomes and treatment-specific effects.

The present study supports the use of circumcision as a beneficial intervention for improving quality of life in male patients with genital LS, particularly in those with phimosis. However, the frequent need to resume topical steroid therapy postoperatively suggests that surgical treatment alone may not suffice for long-term disease control in all patients. Topical therapies remain a cornerstone in managing LS but may be less effective in improving QoL when used in isolation or in patients with severe anatomical complications.

These findings highlight the need for a nuanced, personalised approach to LS management, integrating both medical and surgical options based on disease severity, anatomical involvement, and patient preferences. Future prospective studies with larger cohorts and longer follow-up durations are warranted to better elucidate the optimal treatment algorithms and clarify the role of circumcision in preventing malignant transformation.

## Data Availability

The raw data supporting the conclusions of this article will be made available by the authors, without undue reservation.
